# Diagnosis of Intra-Abdominal Extralobar Pulmonary Sequestration by means of Ultrasound in a Neonate

**DOI:** 10.1155/2013/623102

**Published:** 2013-05-20

**Authors:** Claudio Rodrigues Pires, Adriano Czapkowski, Edward Araujo Júnior, Sebastião Marques Zanforlin Filho

**Affiliations:** ^1^Teaching Center for Computed Tomography, Magnetic Resonance Imaging and Ultrasound (CETRUS), São Paulo, SP, Brazil; ^2^Department of Obstetrics, Federal University of São Paulo (UNIFESP), Rua Carlos Weber, 956 apartamento 113 Visage, Vila Leopoldina, 05303-000 São Paulo, SP, Brazil

## Abstract

Pulmonary sequestration is a congenital abnormality consisting of a mass of pulmonary tissue that presents an abnormal connection with the tracheobronchial tree, with a blood supply coming from an anomalous artery derived from the systemic circulation. Extralobar pulmonary sequestration is characterized by having pleural coverings that are independent of the normal lungs, with vascular supply usually coming from the aorta or from one of its branches. This diagnosis can be suspected prenatally if an abdominal mass, generally below the diaphragm, is seen. Here, we present a case of a neonate on the second day of life, with ultrasonography showing extralobar pulmonary sequestration located above the left adrenal gland that prenatally simulated a neuroblastoma.

## 1. Introduction

Pulmonary sequestration is an uncommon congenital abnormality consisting of a mass of pulmonary tissue that presents an abnormal connection with the tracheobronchial tree, with a blood supply coming from an anomalous artery derived from the systemic circulation. This condition can be classified as extralobar or intralobar, according to its location in relation to the normal lung and its coverings of visceral pleura [1].

Intralobar pulmonary sequestration is characterized by sharing the same visceral pleural coverings that normal lungs have. It is most frequently located in the posterolateral segment of the left lower lobe. In 75% of the cases, its blood supply is derived from the aortic artery, while in the remainder of the cases the blood supply is derived from other thoracic or abdominal vessels. The venous drainage usually takes place through the pulmonary veins. In around 90% of the cases, it is presented as an isolated finding, without other echographic signs [2, 3]. 

Extralobar pulmonary sequestration is characterized by having pleural coverings that are independent of the normal lungs, with vascular supply usually coming from the aorta or from one of its branches. The venous drainage may take place through the azygos, hemiazygos, portal, or pulmonary vein system. The commonest location is the posterior part of the chest, and in 80 to 90% of the cases, it is on the left side. In up to 15% of the cases, it may be located below the diaphragm. In around 60% of the cases, it shows associations with other abnormalities such as congenital diaphragmatic hernia, hydrops, and vertebral and cardiac malformations [3].

Extralobar pulmonary sequestration is generally diagnosed prenatally from the second trimester onwards. On ultrasound, it is usually viewed as a small echodense solid mass that normally has a pyramidal shape; it may or may not contain cystic areas. The commonest locations are the abdomen, where it is often confounded with an adrenal tumor [4], and the extrapulmonary region of the chest [5].

Here, we present a case of extralobar pulmonary sequestration in a neonate in whom prenatal ultrasonography simulated a left adrenal tumor.

## 2. Case Report

**Figure 1:**
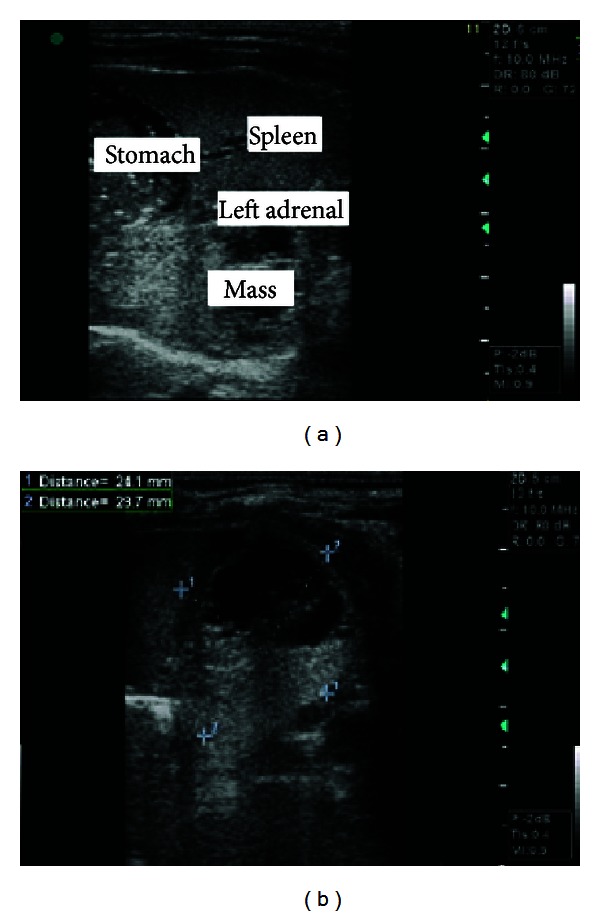
(a) Ultrasonography showing the presence of a retroperitoneal mass, posterior to the left adrenal gland and spleen. (b) Ultrasonography showing the measurements on the mass, of heterogenous appearance with an anechoic formation inside it.

The patient was a female neonate that was born by means of cesarean delivery, with a gestational age of 38 weeks and 2 days and Apgar 9/10. Obstetric ultrasonography was performed in the 32nd week of gestation, and this showed the presence of a left-side adrenal mass suggestive of a neuroblastoma. Postnatal ultrasonography was performed on the second day of life, by means of the Voluson 730 Pro apparatus (GE Medical Systems and Healthcare, Zipf, Austria), with a linear transducer (SP 4–10). This showed the presence of retroperitoneal globular structures located posteriorly to the left adrenal gland, with well-defined edges and a heterogenous interior that was predominantly hyperechogenic but with some anechoic areas inside this, measuring 24.1 × 28.7 × 36.2 mm (Figure 1). Color Doppler was not used for this. The spleen and left adrenal gland did not show any echographic abnormalities and had normal dimensions. No other alterations were seen on the abdominal ultrasonographic examination. 

**Figure 2:**
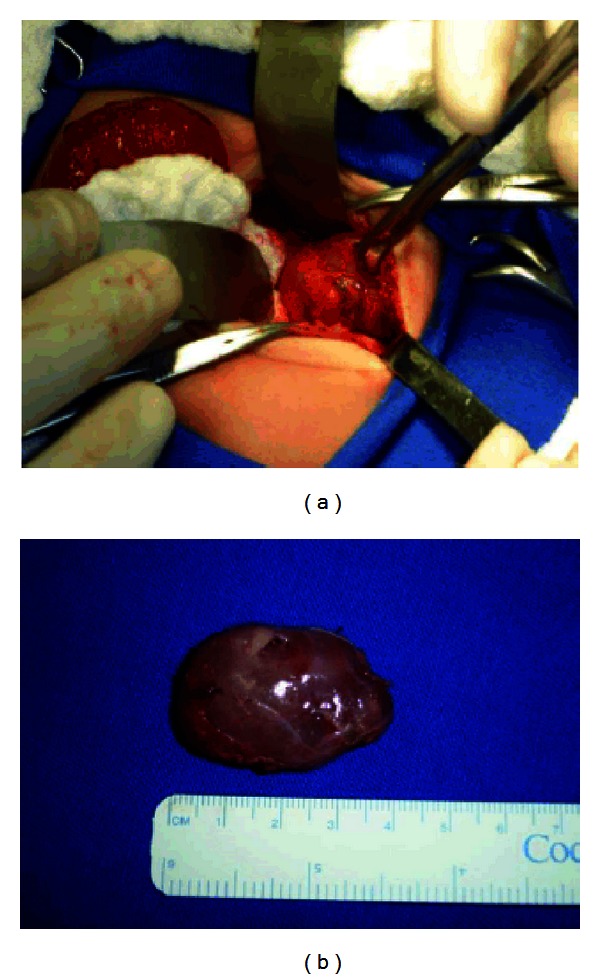
(a) Intraoperative image showing retroperitoneal globular structures of soft consistency. (b) Image of the mass after surgical resection, showing its globular appearance, without any clear feeder vessel.

Surgical treatment was proposed and was carried out on the seventh day of life. This revealed the existence of a solid retroperitoneal formation, of soft consistency, measuring approximately 35 × 25 mm. No feeder vessel was identified. Complete resection was performed and the material was sent for anatomopathological examination, which showed that it was pulmonary tissue (Figure 2). At the six-month followup, the child presented normal growth and development. 

## 3. Discussion

There is no consensus regarding the etiology of pulmonary sequestration, but there are two hypotheses that seem to be more likely: formation and caudal migration of a supernumerary pulmonary bud that accompanies the esophagus and primary pulmonary vascular deficiency, which would explain the persistence of systemic collateral circulation [6]. The distribution between the sexes is similar in cases of the intralobar form, while males predominate in cases of the extralobar form (80% of the cases) [7].

The present study had the aim of highlighting the importance of extralobar pulmonary sequestration among the various differential diagnoses of congenital thoracoabdominal masses. A detailed evaluation on the organs involved is necessary, especially the adrenal glands, with careful analysis of the entire fetal and neonatal morphology, to search for malformations and indicative prognostic signs. The main differential diagnoses are pulmonary cystic adenomatoid malformation, teratomas, neuroblastomas, congenital diaphragmatic hernia, congenital lobar emphysema, laryngeal atresia, pulmonary arteriovenous malformation, and bronchial mucus plug [8]. In our case, the image of the extralobar pulmonary sequestration was confounded prenatally with an adrenal neuroblastoma. Because of the rarity of cases of pulmonary sequestration and the large number of differential diagnoses, the case reported here becomes even rarer given that it involves a female neonate and the extralobar form below the diaphragm.

Although color Doppler was not used in this study, it ought to be used routinely, to complement ultrasonographic examinations so as to attempt to characterize the blood supply to the mass and aid in the diagnosis and surgical planning [9]. Sequestrectomy with careful dissection of the vascular pedicle, which can be done by means of videothoracoscopy, is the preferred surgical treatment [10]. In our case, sequestrectomy was performed by means of a laparotomic approach, with complete removal of the pulmonary tissue. 

Given the rarity of pulmonary sequestration, especially the extralobar form, the present report serves to record the importance of this malformation in the differential diagnosis of congenital thoracoabdominal masses. It also emphasizes the usefulness of pre- and postnatal ultrasonography for diagnosing this condition and for therapeutic planning.
